# Attribute Feature Perturbation-Based Augmentation of SAR Target Data

**DOI:** 10.3390/s24155006

**Published:** 2024-08-02

**Authors:** Rubo Jin, Jianda Cheng, Wei Wang, Huiqiang Zhang, Jun Zhang

**Affiliations:** 1 National Key Laboratory of Science and Technology on Automatic Target Recognition, College of Electronic Science and Technology, National University of Defense Technology, Changsha 410073, China; jinrubo18@nudt.edu.cn (R.J.); zhanghq22@nudt.edu.cn (H.Z.); meiyiwen21@nudt.eud.cn (J.Z.); 2 School of Automation and Electrical Engineering, Linyi University, Linyi 276000, China; chengjianda@lyu.edu.cn

**Keywords:** synthetic aperture radar (SAR) image, data augmentation, feature level, decoupling, capsule neural network (CapsNet), attention mechanism

## Abstract

Large-scale, diverse, and high-quality data are the basis and key to achieving a good generalization of target detection and recognition algorithms based on deep learning. However, the existing methods for the intelligent augmentation of synthetic aperture radar (SAR) images are confronted with several issues, including training instability, inferior image quality, lack of physical interpretability, etc. To solve the above problems, this paper proposes a feature-level SAR target-data augmentation method. First, an enhanced capsule neural network (CapsNet) is proposed and employed for feature extraction, decoupling the attribute information of input data. Moreover, an attention mechanism-based attribute decoupling framework is used, which is beneficial for achieving a more effective representation of features. After that, the decoupled attribute feature, including amplitude, elevation angle, azimuth angle, and shape, can be perturbed to increase the diversity of features. On this basis, the augmentation of SAR target images is realized by reconstructing the perturbed features. In contrast to the augmentation methods using random noise as input, the proposed method realizes the mapping from the input of known distribution to the change in unknown distribution. This mapping method reduces the correlation distance between the input signal and the augmented data, therefore diminishing the demand for training data. In addition, we combine pixel loss and perceptual loss in the reconstruction process, which improves the quality of the augmented SAR data. The evaluation of the real and augmented images is conducted using four assessment metrics. The images generated by this method achieve a peak signal-to-noise ratio (PSNR) of 21.6845, radiometric resolution (RL) of 3.7114, and dynamic range (DR) of 24.0654. The experimental results demonstrate the superior performance of the proposed method.

## 1. Introduction

Synthetic aperture radar (SAR) has been widely applied in both military reconnaissance and civilian observation because of its unique advantages [1,2,3]. Target detection [4] and recognition [5] is an important part of SAR image interpretation, and the algorithms based on deep learning have been rapidly developed and widely used in recent years. However, the training of these models heavily relies on the amount and quality of the target dataset. A complete and rich dataset is indispensable for achieving good generalization of the models. In contrast to optical images, acquiring SAR images through flight tests is more costly and challenging, and the labeling of SAR images is more time-consuming and laborious, so the labeled SAR data for training is quite limited. Additionally, during actual flight tests, the diversity of the acquired SAR image can be constrained due to the special imaging mode of the SAR system.

To resolve these issues, image transformation and target simulation methods are typically employed for data augmentation. As for optical images, methods such as geometric transformation [6], color enhancement [7], adding noise [8], and linear synthesis are often used. However, due to the complexity of the SAR scattering mechanism, these methods do not expand the distribution of datasets. In essence, they are not suitable for SAR image data augmentation. As another important way to obtain SAR images, electromagnetic calculation and simulation technology [9] is relatively low in cost and easy to realize. However, it relies on some prior knowledge, such as the geometric model and material information of the targets. The parameter errors and the electromagnetic calculation errors in the simulation process will make the generated SAR image deviate from the real SAR images to some extent.

With the development of artificial intelligence technology, the method based on deep learning has shown its enormous advantages in SAR image augmentation. This kind of method not only avoids the requirements for experimental equipment and conditions but also breaks through the limitation of prior knowledge and complicated physical modeling. At present, the algorithms widely used for SAR image augmentation mainly include the variational autoencoder (VAE) [10] and generative adversarial network (GAN) [11] models. VAE is a generative network structure based on variational Bayesian inference proposed by Kingma et al. in 2014. The model is divided into two parts, i.e., encoder and decoder. The model predicts the distribution of the latent variable *z*, trains the model with real data to fit the distribution parameters, and then uses the learned model to generate images. Wu et al. use an enhanced VAE to make an initial exploration of the component interpretation of SAR target images [12]. However, for small-sample datasets, VAE cannot achieve the expected results due to the inaccuracy in modeling the data distribution. The training process of VAE becomes unstable because of its variational reasoning, and SAR images generated by VAE are usually blurred since a lot of texture information is lost [13]. In addition, Kullback–Leibler (KL) divergence [14] is introduced to restrict the similarity between potential distribution and prior distribution, and it also limits the diversity and richness of potential representation to some extent.

To solve the problems of the generated images using VAE, Ian Goodfellow et al. put forward a new generation framework, i.e., GAN. This method can establish a model of data distribution [15], and when it is used well with images [16], it can generate clearer and more realistic target images. GAN consists of two parts: generator and discriminator. The generator is mainly used to learn the distribution of real images to make the images generated by itself more realistic, while the discriminator is used to judge whether the received images are true or false. Although GAN has many advantages, it is difficult to train and prone to pattern collapse [17] and gradient disappearance. Models such as deep convolutional generative adversarial network (DCGAN) [18], Wasserstein GAN (WGAN) [19], and Wasserstein GAN using gradient penalty (WGAN-GP) [20] have been put forward to stabilize training and avoid generating meaningless images. However, because the generated image is random and unstable, it is still of little help for SAR target detection and recognition. To address this issue, experts and scholars add conditional information to the input to guide the generation process [21,22,23,24], among which the most typical conditional generative adversarial network (CGAN) incorporates conditional information into both the generator and discriminator, therefore producing SAR target images under specific conditions. Auxiliary classifier GAN (ACGAN) adds an additional classification task between the generator and discriminator, which is beneficial for the generation of more semantic samples. Moreover, information maximizing generative adversarial nets (InfoGAN) [25] proposed by Chen et al. divides the random noise of the generator into two parts to find an interpretable expression: one part is designated as incompressible noise, while the other is characterized as latent variables. Additionally, InfoGAN incorporates a regularization term into the value function of the original GAN model, which is used to compute the mutual information between the latent variables and the output to update both the generator and the discriminator. Although the above methods can solve the problems of unstable training, uncontrollable output, and poor image quality to a certain extent, the training of GAN itself needs many data as support. As a result, these methods still suffer from some problems, such as heavy dependence on data volume, unstable training, and uninterpretable working mechanisms. Among them, interpretability is the key problem that restricts the applicability and efficacy of SAR target-data augmentation. Because of this non-interpretable generative process, it will lead to a contradiction between the authenticity and diversity of the generated images.

To improve the interpretability of GAN-based data-augmentation methods, feature decoupling [26] has been proposed, which refers to separating the changing factors with clear physical meaning or semantic information from real data and giving their corresponding potential representations. These factors are independent of each other. When a single factor changes, it will affect the corresponding content in the generated data, while other information remains unchanged. In the context of CGAN, the generator concatenates conditional information with random noise to produce samples. Meanwhile, the discriminator processes the conditional information and provides feedback to the generator, enabling the generator to adjust the features of the generated samples based on the conditional information. Within the adversarial framework of the CGAN, there is an attempt to decouple the features corresponding to the conditional information. However, the effectiveness of this decoupling is not always optimal. To further improve the decoupling effect, the discriminator of InfoGAN outputs a prediction vector, calculates the mutual information with the input hidden variables, and updates the generator and discriminator according to the mutual information so that more information on hidden variables can be retained in the generated images. Although InfoGAN strives to decouple semantic features such as azimuth and elevation angles by introducing latent codes [27], the relationship between the features and the latent codes still lacks a clear explanation. Under these circumstances, it is only capable of decoupling a singular type of information, and thus, data augmentation can only be conducted based on that specific piece of information about the target. Although the above-improved methods have made efforts to enhance the interpretability, the feature decoupling procedure is carried out under the condition of a “black box”, and the generated images do not follow the scattering mechanisms.

To deal with the aforementioned problems, this paper proposes a feature-level SAR target-data augmentation method by decoupling target attribute feature information and applying targeted perturbations, which enhances the interpretability of SAR data generation. This method maps the feature perturbations of known distribution to target variations of unknown distribution. First, inspired by the decoupling methods such as InfoGAN and capsule neural network (CapsNet) [28], we design a new multi-feature fusion CapsNet as the feature extraction network to fully extract the target features and preliminarily decouple the target attributes. Then, we employ an attention mechanism to further decouple the attribute information, therefore enhancing the orthogonality between attributes. In contrast to the augmentation method using random noise as input, after decoupling the attribute information, we perturb the feature attributes to break through the distribution of the original data, therefore more effectively increasing the diversity of features. Additionally, by substituting SAR images for random signals as input, it is possible to reduce the correlation distance between the input signal and the augmented data, therefore diminishing the demand for training data. In our proposed reconstruction network, we directly compare the original image with the generated image through pixel loss and perceptual loss, which avoids the instability of training and retains more details and structural information. The main contributions of this study are summarized as follows:

(1) We propose a feature extraction framework based on multi-feature fusion CapsNet, which can effectively decouple target attributes. Furthermore, an attention mechanism is implemented to achieve a further decoupling of target features, therefore enhancing the interpretability of SAR target-data augmentation;
(2) In the process of image reconstruction, we integrate pixel loss and perceptual loss to construct a joint loss function that constrains the generation procedure. This approach not only enhances the quality and reliability of the generated images but also increases the stability of the model training by directly comparing the original images with the generated ones;
(3) We propose a mapping approach from feature perturbations of known distribution to target variations of unknown distribution. This approach substitutes real images for random signals to achieve more stable and reliable feature-level data augmentation. Moreover, it can achieve more effective learning with a smaller volume of data and is more suitable for small-sample SAR target data.

The remainder of this paper is organized as follows. Section 2 elaborates on the theoretical foundations of CapsNet. The methodology is presented in Section 3 in detail. Experimental results and discussions are provided in Section 4. Section 5 concludes the paper.

## 2. Basic Theory of CapsNet

It is well-known that convolutional neural networks (CNNs) [29], widely used in deep learning, perform well on image data-related problems, but they also have significant limitations. The pooling layers in CNNs retain the largest activation value, which is the most prominent feature, during each pooling operation. As a result, even if there are changes in the pixels of the image, the pooling layer may fail to perceive them, leading to the loss of a substantial amount of valuable information during the pooling process. Moreover, the neurons in one layer of a CNN pass scalar values to the neurons in the next layer, lacking directionality, which cannot represent the spatial relationships between high-level and low-level features, as well as the spatial relationships between low-level objects. This leads to CNNs’ inability to effectively learn the spatial relationships between target features. Using brute force methods to learn the structural information of targets inevitably requires a large dataset, but this approach, while offering limited improvement, does not fundamentally solve the problem. Therefore, Hinton, G.E. et al. [28] proposed CapsNet as a powerful alternative to CNNs. They are based on a new structure—the neural capsule, which uses a vector composed of a group of neurons instead of individual neurons, capable of learning more invariant image representations. Additionally, the dynamic routing mechanism in CapsNet makes them more robust to changes in the pose and spatial relationships of parts of objects in images. CapsNet can address the issues of CNNs, such as the need for many training samples due to the inability to recognize the pose of images, weak feature representation capabilities, and the loss of spatial information due to pooling operations.

**Figure 1:**
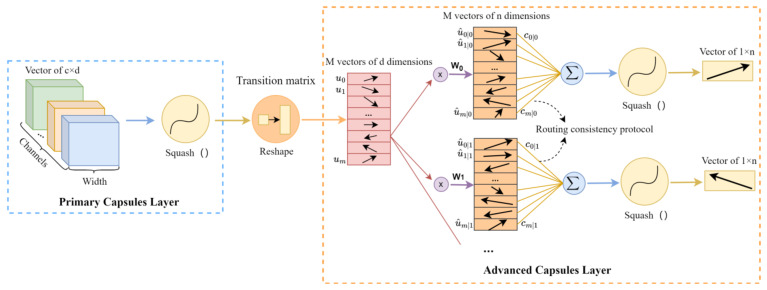
Schematic diagram of advanced capsules.

The initial CapsNet generally consists of four layers: an input layer, two convolutional layers, and a fully connected layer [30]. The first layer is the input layer. The second layer is a convolutional layer with a larger kernel size to achieve a larger receptive field. The third layer is a convolutional layer known as the primary capsule layer. In this layer, the subjects of the convolutional operation are not individual neurons but larger neural capsules. The number of output channels in this layer will be reduced, but each channel contains multiple convolutional kernels to extract multiple features. Within each channel, multiple features form a vector that encapsulates multiple attributes into a primary capsule. The final layer is the advanced capsule layer, which is a fully connected layer. To adapt the convolutional layers to the subsequent fully connected layer, there is an additional process of reshaping the data matrix dimensions between the primary capsules layer and the advanced capsules layer. A schematic diagram is shown in Figure 1.

The dynamic routing mechanism used for the connections between the primary capsules layer and advanced capsules layer in a CapsNet ensures that the output of a capsule is only sent to the appropriate parent nodes. In CapsNet, the coupling coefficient between the primary capsule *i* and the advanced capsule *j* is evaluated by a scalar weight *c_ij_*, which indicates how the primary capsule sends its output vector to the advanced capsule. The core work of the dynamic routing part is to find the best weight value *c_ij_*. The weight value *c_ij_* is the processing result of *b_ij_* under the SoftMax function, as shown below.

(1) $$c_{ij}=\frac{\exp(b_{ij})}{\sum_{k}\exp(b_{ik})}$$

**Figure 2:**
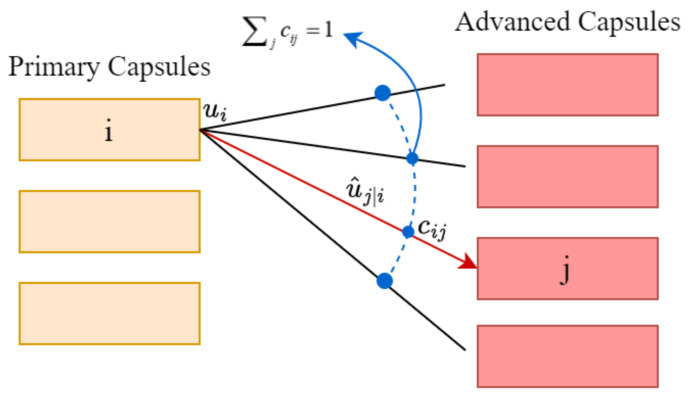
Schematic diagram of dynamic routing mechanism.

The dynamic routing algorithm repeats operations in each round: A. Normalization; B. Forecast output; C. Weighted summation; D. Compressing the vector; E. Update the weights. Then, return to Step A and repeat several rounds. The schematic diagram of the dynamic routing mechanism is shown in Figure 2.

In the dynamic routing mechanism, the process of transferring the primary capsule to the advanced capsule is defined as

(2) $$s_{j}=\sum_{i}c_{ij}{\overset{\wedge }{u}}_{j|i}$$

Among them, ${\overset{\wedge }{u}}_{j|i}$ is the prediction vector of primary capsule *i* to advanced capsule *j*, and *c_ij_* is the corresponding coupling coefficient. The prediction vector is obtained by multiplying the output *u_i_* of the primary capsule itself by the weight matrix *W_ij_*, i.e.,

(3) $${\overset{\wedge }{u}}_{j|i}=W_{ij}u_{i}$$

Generally speaking, the advanced capsules are much larger in size and contain more information than the primary capsules. The routing mechanism through this protocol is more effective than the original routing form realized by maximum pooling because, in addition to retaining the most active feature detectors in the local area, the next-level capsules are selectively ignored.

CapsNet has currently gained widespread consensus for its efficacy in feature extraction [31,32]. Moreover, they can achieve feature decoupling. Consequently, this paper utilizes CapsNet for the extraction of features.

## 3. Methodology

### 3.1. Overall Framework

**Figure 3:**
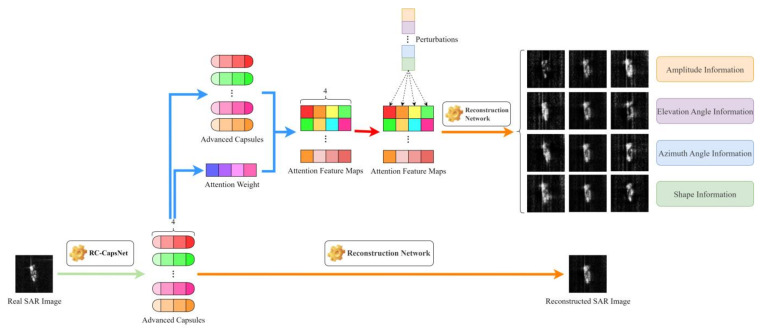
Overall architecture.

Figure 3 shows the overall architecture of the proposed method. This method includes three steps: feature extraction based on residual connected CapsNet (RC-CapsNet), attribute decoupling based on attention mechanism, and image reconstruction after feature-level perturbations. First, an improved CapsNet is employed to capture target features that possess significant spatial positional relationships and to achieve preliminary decoupling of target features, obtaining advanced capsules with attribute information. Subsequently, an attention mechanism is applied to the advanced capsules from which attribute information has been decoupled to integrate along the attribute dimensions and enhance the orthogonality between attributes, therefore achieving further decoupling of target attributes. Finally, perturbations from known distributions are incrementally applied to the feature dimensions corresponding to the target attributes, and the image is reconstructed through a reconstruction network, which allows the distribution of the original data to be transcended, yielding augmented images at the feature level for SAR targets. In this section, we will introduce the details of these three parts.

### 3.2. Feature Extraction Based on RC-CapsNet

The foundation of CapsNet lies in the concept of neural capsules, which was originally proposed by Hinton, G.E. et al. [28] as a method for learning robust, unsupervised representations of images. A neural capsule is a group of neurons that are locally invariant, encapsulating various properties that determine the existence of an entity. The output of a capsule is a high-dimensional activity vector, where the magnitude of the vector, after normalization, can represent the probability of the entity’s existence. The direction of the vector characterizes the parameters of the corresponding entity, which Hinton refers to as “pose,” encompassing attributes such as position, orientation, scale, and color.

To ensure that the magnitude of the capsule’s output vector can adequately represent the model of entity existence, we apply a nonlinear squashing function that constrains the length of the output vector to a maximum of 1. This approach not only maintains the direction of the vector but also compresses the magnitude value into the interval [0, 1). The formula for this compression function is as follows.

(4) $$v_{j}=\frac{{\left\|s_{j}\right\|}^{2}}{1+{\left\|s_{j}\right\|}^{2}}\cdot \frac{s_{j}}{\left\|s_{j}\right\|}=\frac{{\left\|s_{j}\right\|}^{2}}{1+{\left\|s_{j}\right\|}^{2}}{\overset{\wedge }{s}}_{j}$$

where *v_j_* is the output vector of the compression function and *s_j_* is the output vector. $\frac{{\left\|s_{j}\right\|}^{2}}{1+{\left\|s_{j}\right\|}^{2}}$ is the proportion of the scaling vector, and $\frac{{\left\|s_{j}\right\|}^{2}}{1+{\left\|s_{j}\right\|}^{2}}{\overset{\wedge }{s}}_{j}$ is the reserved unitized vector.

**Figure 4:**
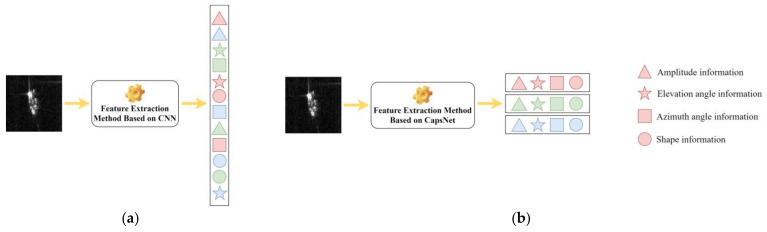
Comparison of two feature extraction methods. (**a**) Feature extraction method based on CNN; (**b**) Feature extraction method based on CapsNet.

Due to the loss of substantial target information, feature extraction methods based on CNNs are not sensitive to transformations such as translation, rotation, and scaling of objects. This mode of extraction also results in the blending of various types of information pertaining to the target. In contrast, feature extraction methods based on CapsNet encapsulate multiple neurons into neuron capsules, which can express multiple pieces of information simultaneously and relatively independently. More importantly, this CapsNet-based feature extraction method can achieve a degree of decoupling of target features. Figure 4 illustrates the distinction between the two types, where each row in the right-hand diagram represents a capsule containing various attribute information, and each column represents a feature representation of a specific attribute. In this paper, the information decoupled from SAR target-data features is categorized into four attributes: intensity, elevation angle, azimuth angle, and shape.

**Figure 5:**
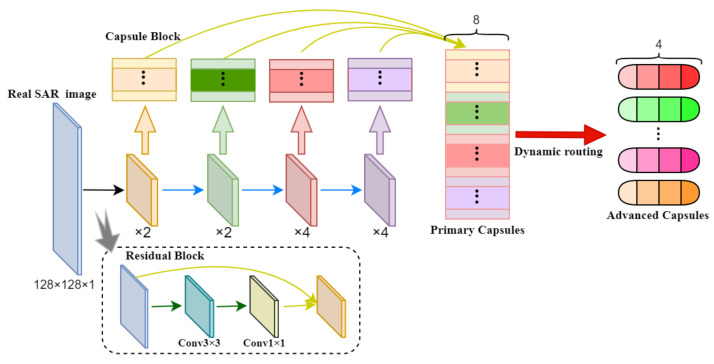
Structure diagram of RC-CapsNet.

Despite the numerous advantages of CapsNet, traditional CapsNet also suffers from issues such as simplistic network architecture, lack of connections between different layers of the multi-layer network structure [33], and insufficient feature extraction capabilities. Inspired by the convolutional networks with residual connections (ResNet) [34] and the cross-stage partial networks (CSPNet) [35], we design a novel method to enhance the feature extraction capabilities of CapsNet, with the network structure depicted in Figure 5. Initially, real SAR images are taken as the input to the network and undergo feature extraction through multiple residual blocks, which is achieved through different channels. Subsequently, the features extracted from the same layer with varying attributes are encapsulated into a capsule block. Four such capsule blocks are stacked and fused to form primary capsules. Finally, the primary capsules are transformed into advanced capsules that encompass rich feature information through a dynamic routing mechanism.

The network architecture proposed in this method enhances the feature extraction capabilities of CapsNet through a multi-level, multi-channel approach for target feature extraction. Furthermore, with our improvements, each convolutional layer in the network model we propose can directly connect to primary capsules. As a result, both high-level and low-level feature information can be directly fed into the primary capsules. More advantageously, the loss from the primary capsule layer can directly reach each convolutional layer, significantly reducing the backpropagation distance. Additionally, the backpropagation distance does not increase with the number of convolutional layers, thus avoiding the issue of reduced network convergence performance due to increased backpropagation distance [36]. This addresses the problem of deep network hierarchies requiring extensive training data, allowing for satisfactory feature extraction effects even with limited data.

### 3.3. Attribute Decoupling Based on Attention Mechanism

**Figure 6:**
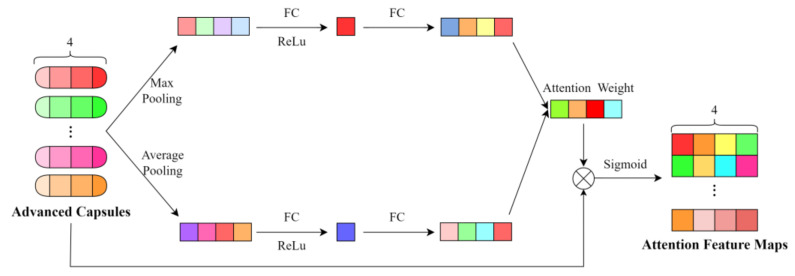
Structure diagram of attention network.

Merely employing residual connections within CapsNet does not adequately decouple feature information. Therefore, to further decouple feature information, we introduce an attention mechanism with the network architecture depicted in Figure 6. Initially, we perform max pooling and average pooling operations on the advanced capsules obtained from feature extraction for each attribute, calculating the maximum and average feature values for each attribute, which represent the most salient and average features of the attribute, respectively. Subsequently, the maximum feature vectors and average feature vectors are passed through a fully connected layer and then summed to obtain the attention weights. By multiplying these attention weights with each attribute of the original feature map, we obtain an attention-weighted feature map that emphasizes the significance of each attribute.

By employing an attention mechanism, we further integrate the attribute information and enhance the orthogonality between different attribute features, therefore obtaining target features that are more interpretable.

### 3.4. Image Reconstruction after Feature-Level Perturbations

**Figure 7:**
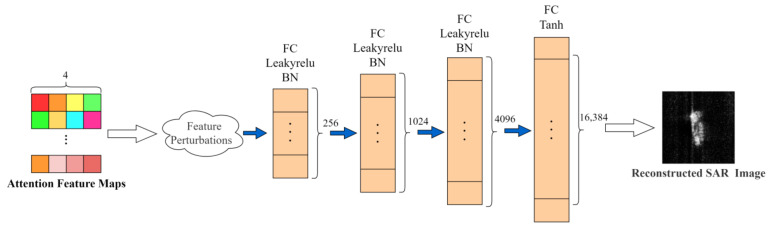
Image reconstruction process.

As can be seen from Section 2, the target features are decoupled into four attributes: amplitude, elevation angle, azimuth angle, and shape. To validate this conclusion, we reconstruct the target features into an image while applying individual perturbations to the features of each attribute during the reconstruction process, with the network structure for reconstruction depicted in Figure 7. Initially, we impose perturbations on the attention-weighted feature map along a specific attribute, which is then processed through four fully connected layers to obtain the reconstruction SAR target image.

**Figure 8:**
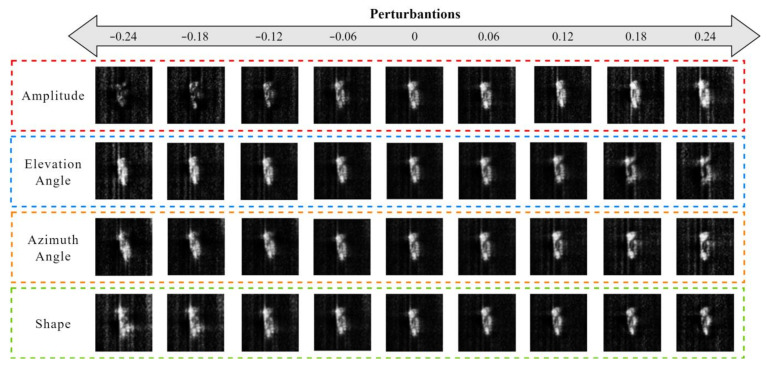
Results of image reconstruction under different perturbations.

In the columns corresponding to different attributes of the target features, we apply perturbations within the range of [−0.24, 0.24] with an interval of 0.06. As illustrated in Figure 8, when perturbations are applied to the column corresponding to the intensity attribute, the scattering intensity of the reconstruction SAR target undergoes overall changes. When perturbations are applied to the column corresponding to the elevation angle attribute, the scattering points of the reconstruction SAR target change, along with local scattering intensity variations. When perturbations are applied to the column corresponding to the azimuth angle attribute, the overall orientation of the reconstruction SAR target gradually shifts. When perturbations are applied to the column corresponding to the shape attribute, the structural information of the reconstruction SAR target undergoes changes to varying degrees.

The aforementioned experimental results not only validate the four types of decoupled attribute information but also demonstrate that this method can achieve the augmentation of specific features in SAR target data, offering a novel perspective for SAR image augmentation methods. In the data-augmentation process of this method, perturbations are applied at the feature level. Hence, the variation factors are not at the input level but at the feature level. This approach substitutes feature errors for element-wise errors and provides invariance during the variation process.

### 3.5. Augmentation Process and Loss Function

During the network training process, we initially input real SAR images into the RC-CapsNet, subsequently feed the extracted features into the attention network, and ultimately obtain the augmented SAR target imagery through the reconstruction network. In the process of augmenting the SAR target image data, we utilize the trained network to apply perturbations to features that have undergone decoupling to acquire attribute information. For instance, to augment SAR target image data of different azimuth angles, we apply perturbations to the column of features corresponding to azimuth angle information output by the attention mechanism. Inspired by VAE and GAN, the perturbations we apply during the augmentation process are noise that conforms to a normal distribution. Ultimately, after applying perturbations to the features, we reconstruct the SAR target information, therefore obtaining augmented images of SAR targets that are tailored to specific features.

Thus, by applying perturbations and reconstructing specific attributes, we can obtain SAR target augmentation data with varying amplitudes, elevation angles, azimuth angles, and shapes.

To better train the network, we jointly use two loss functions during the training process: mean squared error (*MSE*) and learnable perceptual image patch similarity (LPIPS) [37]. The mean squared error penalizes outliers exponentially at the pixel level, enabling the reconstruction of images with the calculation formula as follows.

(5) $$MSE(x_{r},x_{o})=\frac{1}{m}\sum_{i=1}^{m}{\left(x_{r}-x_{o}\right)}^{2}$$

where *x_r_* is the reconstruction image data, *x_o_* is the original image data, and *m* is the number of pixel values in the image.

**Figure 9:**
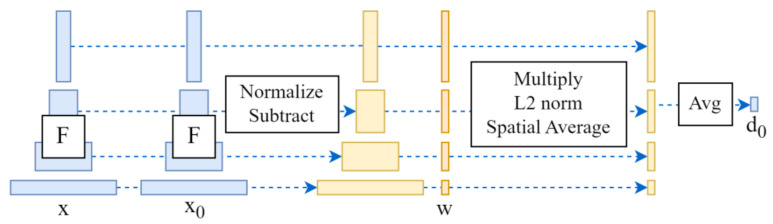
LPIPS calculation process.

However, the exclusive use of *MSE* can lead to issues of image smoothing and blurring. Through our literature review and experimental research, we find that the LPIPS metric can address, to some extent, the issue of evaluation failure caused by image smoothing when employed as an image quality assessment tool. Therefore, we incorporate the LPIPS into our loss function. LPIPS, introduced by Zhang, R., et al. in 2018, [37] is utilized to measure the difference between two images. This metric learns the inverse mapping from generated images to ground truth, enforcing the generator to learn the inverse mapping from fake images back to real images and addresses the perceptual similarity between them. LPIPS aligns more closely with human perception compared to traditional methods (such as L2, peak signal-to-noise ratio (PSNR) [38], structural similarity index measure (SSIM) [39], feature similarity index measure (FSIM) [40], etc.). For smooth images, traditional evaluation methods are likely to fail, and currently, generative models like GAN, especially VAE, tend to produce overly smooth results. LPIPS employs a deep learning model to learn feature representations of images, which can better capture high-level semantic information of images and, to some extent, resolve the issue of focusing exclusively on superficial information during image reconstruction. Moreover, the approach of computing image similarity by dividing the image into patches and comparing them can help understand differences in various regions of the image, therefore complementing the attention to local information to a certain degree and enhancing the interpretability of feature representations. Leveraging these advantages, we incorporate LPIPS as a loss function and combine it with MSE to constrain the reconstruction process. LPIPS extracts features through a neural network and calculates the differences between these features, with its working principle illustrated in Figure 9.

LPIPS sends two inputs, *x_o_* and *x_r_* to neural network F for feature extraction. After activation, the output of each layer is normalized and recorded as ${\overset{\wedge }{y}}^{l},{\overset{\wedge }{y_{o}}}^{l}\in R^{H_{l}\times W_{l}\times C_{l}}$. Then, the L2 distance is calculated after point multiplication with the W-layer weight, and finally, the average distance is obtained. The formula is as follows:

(6) $$d(x_{r},x_{o})=\sum_{l}\frac{1}{H_{l}W_{l}}{\left\|w_{l}⊕({\overset{\wedge }{y}}_{hw}^{l}-{\overset{\wedge }{y}}_{ohw}^{l})\right\|}_{2}^{2}$$

The weights of the two loss functions are hyperparameters set as *α* and *γ*, respectively. *α* is a hyperparameter representing the weight of the MSE, which describes the proportion of pixel loss in the overall loss function and is used to control the importance of pixel loss in the reconstruction process. By adjusting *α* and *γ*, the model’s sensitivity to the pixel level of the image during the reconstruction process can be controlled, therefore helping the model to achieve a balance between pixel and perceptual levels. By normalization, we set the weight for the MSE to 1 and the weight for the LPIPS to *β*. Hence, the final loss function we utilize is

(7) $$\mathrm{Loss}=\frac{1}{m}\sum_{i=1}^{m}{\left(x_{r}-x_{o}\right)}^{2}+\beta \cdot \sum_{l}\frac{1}{H_{l}W_{l}}\sum_{h,w}{\left\|w_{l}⊕({\overset{\wedge }{y}}_{rhw}^{l}-{\overset{\wedge }{y}}_{ohw}^{l})\right\|}_{2}^{2}$$

## 4. Experiments and Discussions

### 4.1. Data and Settings

#### 4.1.1. Description of Dataset

In this paper, we use the vehicle target image set collected by Ku-band SAR carried by UAV to verify the effectiveness of the proposed method.

**Figure 10:**
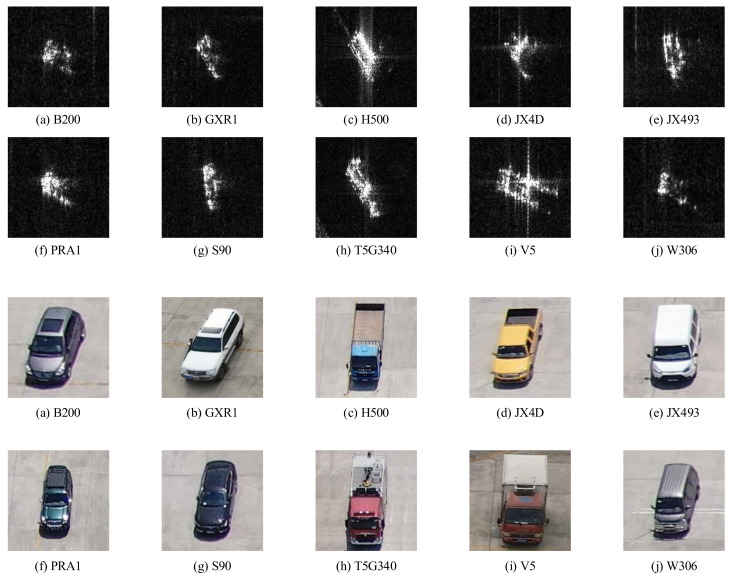
SAR and optical images of ten kinds of vehicle targets.

The image set consists of ten kinds of vehicle targets, and the image resolution is 0.15m. During the experimental process, we exclusively utilized data under the VV polarization condition. Figure 10 shows the SAR vehicle target images under VV polarization and the optical images of the corresponding type of vehicle. Among them, B200 is a Mercedes-Benz sedan, GXR1 is a Toyota SUV, H500 is a blue low-hurdle truck, JX4D is a yellow pickup truck, JX493 is a white Tushun commercial vehicle, PRA1 is a Prado SUV, S90 is a Volvo sedan, T5G340 is a fire truck, V5 is a red van, and W306 is a gray commercial vehicle.

Each category contains SAR images with elevation angles of 25°, 30°, and 45°, respectively, with azimuth angles ranging from 0° to 360° and intervals of 5°. Due to the corruption of the imaging data, there are individual missing images for each elevation angle. Thus, for each vehicle category, there are 71 images captured at a pitch angle of 25°, 71 images at a pitch angle of 30°, and 70 images at a pitch angle of 45°, accumulating to a total of 2120 images.

#### 4.1.2. Parameter Setting

In our experiments, to more effectively demonstrate the generative performance of our method under conditions of limited datasets, we only use the data of 30° and 45° elevation angles to augment the data, and all the images are cropped to 128 × 128 without any other processing. The range of random perturbation in the process of image generation is [−0.3, 0.3]. Use “Adam” as the optimizer and set the learning rate to 0.0002. The exponential decay rate of first-order moment estimation is set to 0.5, and the exponential decay rate of second-order moment estimation is set to 0.999. The batch size is set to 8. The weight value *β* of the loss function is set to 0.01. A total of 400 training epochs are conducted. 

In our experiments, the operating system is Ubuntu 18.04.6 LTS, the information of CPU is Intel(R) Core (TM) i9-10940 CPU @ 3.30GHz, the information of GPU is Nvidia GeForce RTX 3090, the video memory is 24 G, the version of CUDA is 11.4. The frame is pytorch, the version of torch is 1.12.0, and the version of torchvision is 0.13.0.

### 4.2. Image Quality Evaluation

In this section, we conduct data augmentation using VAE, DCGAN, variational autoencoder generative adversarial network (VAE-GAN) [41], and MCGAN [42], as well as our proposed method. Despite the existence of numerous other image generation methods, only a small part of them have been utilized for SAR image generation. Moreover, these methods are prone to mode collapse when the data are inadequate, which can lead to training infeasibility. Therefore, we chose the aforementioned five methods for comparative analysis.

#### 4.2.1. Qualitative Evaluation

**Figure 11:**
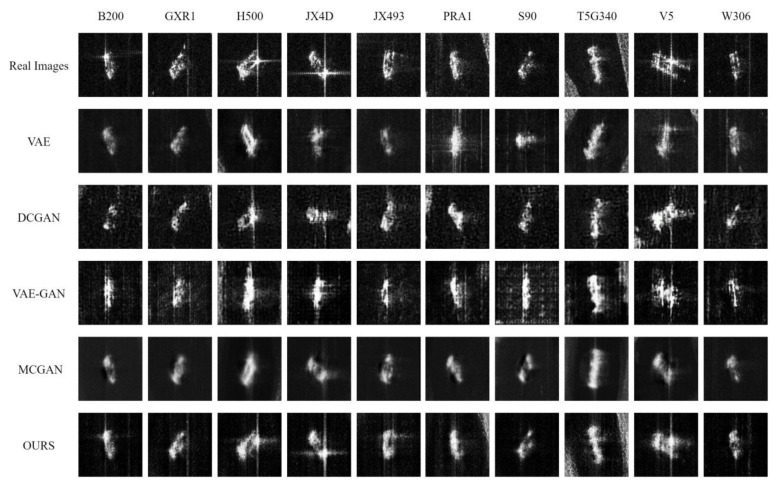
Comparison between real SAR images and various augmented images.

We first describe the intuitive evaluation of generated data by human vision, and the results generated by different networks are presented in Figure 11, together with the real images.

In the figure above, the rows correspond to different generation methods, while the columns correspond to targets of various categories. The first row contains images of real SAR vehicle targets.

From the images in the second row, it can be observed that while the VAE can learn the contour information of SAR target images relatively completely, the overall generated images are somewhat blurred. They retain only the edge textures, with the internal texture details being entirely smoothed out, exhibiting a characteristic of over-smoothing. Notably, the augmented images for the B200, GXR1, JX4D, and JX493 vehicle targets are particularly indistinct, with more texture information lost. In particular, the JX4D and V5 vehicle targets can no longer be accurately generated. In contrast, the augmented images for the H500, PRA1, and T5G340 vehicle targets are relatively less blurred and retain some texture information.

From the images in the third row, it is evident that the DCGAN is more capable of capturing the complex distribution and details of the input data. The generated SAR images exhibit more distinct texture details, but they have significant background noise, and the contours of the targets are also quite chaotic. Additionally, the structure of the generated images differs greatly from the actual images, not conforming to the scattering characteristics of real SAR targets. The augmented images for the GXR1 and T5G340 vehicle targets are particularly irregular in structure, with shapes that are clearly inconsistent with reality. Moreover, the background noise for the JX4D and V5 vehicle targets is very prominent, severely affecting the normal interpretation of SAR target images. However, it shows good results for H500 and JX493 vehicle targets.

From the images in the fourth row, it can be seen that the VAE-GAN, which combines the concepts of VAE and GAN, can more comprehensively capture the structure and edge information of SAR targets. The generated images have more complete structural information, more pronounced amplitude information, and scattering points that better conform to the scattering mechanism of SAR targets. However, the background noise is also noticeable, and it appears the checkerboard artifacts [43], along with some degree of mode collapse. It shows better results for H500, T5G340, and V5 targets but suffers from mode collapse with other targets.

From the images in the fourth row, it can be seen that while the images generated by MCGAN can learn the overall contour information of SAR target images, the generated images are also relatively blurred. Due to the small size of the existing training dataset, the network cannot effectively learn the internal information of the target images. Unlike VAE, not only are the internal texture details of the target images smoothed out, but the background noise is also smoothed. The vehicle target W306 has lost most of its information. In contrast, the JX4D, JX493, and V5 vehicle targets have retained some textural information.

By comparison, it can be seen that our proposed method achieves the best generation performance. As shown in the fourth row, the images generated by our method not only present texture and edge information but also have a cleaner background without the phenomenon of mode collapse. Most importantly, the generated images have very distinct strong and weak scattering points, which are in line with the scattering characteristics of SAR targets, offering strong reliability and being more conducive to the interpretation of SAR images.

#### 4.2.2. Quantitative Evaluation

Additionally, we conducted a quantitative comparison using three evaluation metrics: PSNR, radiometric resolution (RL) [44], and dynamic range (DR). PSNR is an engineering term that represents the ratio of the maximum possible power of a signal to the power of the noise affecting its representation, and a higher PSNR indicates better image quality and lower noise. RL reflects the ability of SAR images to distinguish the backscattering coefficients between targets and is used to assess the grayscale resolution of the image. DR is the ratio of the maximum to the minimum grayscale values in an image. In engineering, the dynamic range of SAR images is typically defined as the ratio of the average grayscale of the target area to the average grayscale of the background area. A larger dynamic range indicates a greater difference in pixel grayscale between the target and the background, resulting in higher contrast.

We randomly selected 50 original images with the elevation angles of 30° and 45° mixed, and for each of the implementing methods, we randomly selected 50 images from the generated methods. We then calculated their respective statistical values, which are presented in Table 1.

In Table 1, a comparison of the image metrics generated by various methods reveals that our method outperforms the others in all metrics except for DR. Since the real images are devoid of distortion, we do not compute the PSNR for them. From the perspective of the PSNR metric, it can be observed that the images generated by our method have the highest value, indicating less image distortion, better quality, and lower noise. Among the other four methods, the VAE exhibits a slightly higher PSNR due to the cleaner background and less noise in the generated images, resulting in less distortion during the calculation process. Among the other four methods, the VAE-GAN is more adept at learning the structural information of the target image. Regarding RL, the images generated by our method have a higher resolution, indicating more detailed content, whereas the images generated by VAE, which lost many details due to blurring, have a lower radiometric resolution. Due to the scarcity of training samples, the images generated by MCGAN exhibit relatively poor performance across all metrics except for DR.

In summary, both qualitative evaluation and qualitative evaluation indicate that our method yields better results, demonstrating its ability to generate higher-quality augmented images.

### 4.3. Discussion

#### 4.3.1. Ablation Study

In this paper, we further carry out ablation experiments for the proposed method to analyze the performance of each module of the network. First, to verify the validity of the residual connecting block in the CapsNet, we compare the results generated by the proposed method and traditional CapsNet. The PSNR evaluates the overall reconstruction quality of the augmented images, and the RL assesses the quantitative precision of the details within the augmented images. Therefore, these two metrics can provide a more accurate and representative quality evaluation. In this experiment, we select H500 and V5 targets as the augmented targets and take PSNR and RL as the evaluation indexes. The results are shown in Table 2.

We compare the images reconstructed by two different methods quantitatively. As can be seen from Table 2, the images corresponding to the traditional CapsNet are worse than those of RC-CapsNet in terms of PSNR and RL indexes. This is because the traditional CapsNet has a simple structure and insufficient ability to extract features, which leads to the fact that the spatial position relationship of the target cannot be well learned, and its ability to decouple features is also limited. Therefore, it is not possible to effectively reconstruct the target structure and detailed information, which in turn affects the performance of image generation. Our method employs a multi-layer residual connection architecture, which not only effectively enhances the capability of feature extraction but also integrates features extracted at different levels. This approach more completely preserves the feature information present in the original image. Consequently, both the reconstruction quality and the detail precision of the generated image are improved.

We continue to analyze the effectiveness of the attention mechanism. We compare the results generated by the proposed method with and without the attention mechanism, and the computed indexes are shown in Table 3.

It can be seen from Table 3 that the two indexes of the images generated by the method without attention mechanism are slightly worse than those of our method because only the improved CapsNet cannot adequately decouple the target features. There is still entanglement between the target attribute features. Employing the attention mechanism can further enhance and separate the features, strengthen the correlation among features of the same attribute, and enhance the orthogonality between different attributes. Therefore, during the reconstruction process, the features can be reconstructed more selectively, resulting in higher-quality images.

#### 4.3.2. Determination of the Weight of the Loss Function

**Figure 12:**
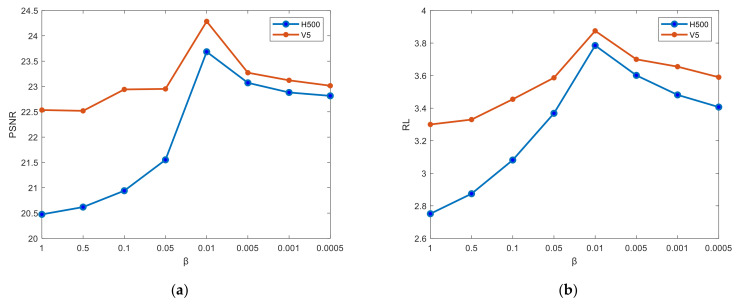
Relationship between different evaluation index and *β* of H500 and V5 targets augmented images. (**a**) PSNR; (**b**) RL.

In the training process, our loss function is Equation (7), where *β* is the hyperparameter, which is used to control the relative weight of pixel loss and perceptual loss during the reconstruction process. In this regard, we do many experiments to find the best weight. During the experimental process, we performed data augmentation using different weight ratios *β*. Then, we analyzed the PSNR and RL of the generated images to find the optimal weight values. The results are shown in Figure 12.

Through the two sets of experiments mentioned above, it can be observed that when the value of *β* is set to 0.01, both the PSNR and RL reach their maximum values. Consequently, under this weight ratio, the quality of the generated images is the best, which implies that the performance of the image generation model is also at its optimum.

#### 4.3.3. Computational Complexity Analysis

Computational complexity is an important metric for evaluating an algorithm. The assessment of computational complexity generally includes time complexity and space complexity.

Time complexity determines the training or prediction time of the model. If the complexity is too high, it will lead to a significant amount of time being consumed for model training and prediction. We typically measure time complexity by the number of operations performed by the model, using FLOPs (floating point operations per second) as the evaluation metric. According to our calculations, the time complexity of the model we propose is 7.87 GFLOPs.

Space complexity determines the number of parameters in the model. The more parameters the model has, the greater the amount of data required for training. We typically measure space complexity by the number of parameters and the size of the model storage, using total parameters and parameter size as the evaluation metrics. According to our calculations, the total parameters of the model we propose is 91,642,832, and the parameter size is 349.59 MB. This is mainly due to the dynamic routing mechanism in the capsule network model, which greatly increases the number of model parameters.

Based on the analysis of the aforementioned computational complexity, both the space complexity and time complexity of our model are relatively high, but they are within an acceptable range.

## 5. Conclusions

In this paper, we propose a feature-level data-augmentation method for SAR target images. We decouple the attribute features of targets through an improved CapsNet and an attention mechanism-based framework, improving the effect of feature extraction and increasing the interpretability of the network. For the decoupled attribute information, we validate its reliability and apply perturbations to realize data augmentation. Unlike previous methods, the proposed augmentation approach learns the transformation from known distributions to unknown distributions. The variability of this method is not at the input end but at the feature level. This shortens the association distance from input signals to augmented data, therefore providing stronger generalizability. In response to the training challenges and data dependency issues of modern neural networks due to their imperfections, the proposed method has successfully addressed these issues. Additionally, the dual constraints of pixel loss and perceptual loss effectively enhance the quality of image generation. Extensive experiments demonstrate the feasibility and effectiveness of our method. The proposed method not only provides a new perspective for the effective augmentation of SAR target data but also offers superior performance in terms of PSNR, RL, and DR. In practical applications, due to the inherent sensitivity of the capsule network to object posture, it focuses more on the target itself and, therefore, exhibits strong robustness against background interference. However, the computational complexity of this method is relatively high, leading to higher hardware costs and energy consumption, making it difficult to deploy on resource-constrained device platforms. Furthermore, although the proposed method follows the scattering characteristics of SAR targets, the generation results are not yet clear enough. In the next, we intend to integrate target structural information into the generation process to further improve the quality of the generated SAR images and, additionally, to conduct further research on the lightweight design of the model.

## Figures and Tables

**Table 1:** Parameters of SAR original images and generated images.

Evaluation Index	PSNR	RL	DR
Real images	-	3.7450	24.0654
VAE	18.1154	3.1166	20.8279
DCGAN	17.3358	3.5883	24.0654
VAE-GAN	17.5197	3.5952	24.0654
MCGAN	16.1487	3.0059	24.0654
OURS	21.6845	3.7114	24.0654

**Table 2:** Performance comparison of two kinds of CapsNet of H500 and V5 targets.

Target	Evaluation Index	CapsNet	OURS
H500	PSNR	21.3543	22.4563
	RL	3.4431	3.7198
V5	PSNR	21.7648	23.6577
	RL	3.3195	3.8494

**Table 3:** Performance comparison of attention mechanism with or without attention mechanism of H500 and V5 targets.

Target	Evaluation Index	Without Attention Mechanism	OURS
H500	PSNR	20.2335	22.4563
	RL	3.5168	3.7198
V5	PSNR	21.1681	23.6577
	RL	3.3137	3.8494

## Data Availability

The data are not publicly available due to privacy.
